# Lomefloxacinium picrate

**DOI:** 10.1107/S1600536811002534

**Published:** 2011-01-26

**Authors:** Jerry P. Jasinski, Ray J. Butcher, M. S. Siddegowda, H. S. Yathirajan, Q. N. M. Hakim Al-arique

**Affiliations:** a Department of Chemistry, Keene State College, 229 Main Street, Keene, NH 03435-2001, USA; b Department of Chemistry, Howard University, 525 College Street NW, Washington, DC 20059, USA; c Department of Studies in Chemistry, University of Mysore, Manasagangotri, Mysore 570 006, India

## Abstract

In the cation of the title compound [systematic name: (*RS*)-4-(3-carb­oxy-1-ethyl-6,8-difluoro-4-oxo-1,4-dihydro­quinolin-7-yl)-2-methyl­piperazin-1-ium 2,4,6-trinitro­phenolate], C_17_H_20_F_2_N_3_O_3_^+^·C_6_H_2_N_3_O_7_^−^, the piper­azine ring adopts a slightly distorted chair conformation and contains a protonated N atom. An intra­molecular O—H⋯O hydrogen bond occurs in the cation. The dihedral angles between the mean planes of the six-atom piperazine ring and the 10-atom fused ring system is 43.3 (5)°. The picrate anion inter­acts with the protonated N atom of an adjacent cation through a bifurcated N—H⋯(O,O) three-center hydrogen bond. Strong N—H⋯O hydrogen bonds in concert with weak π–π stacking inter­actions [centroid–centroid distance = 3.6460 (14) Å] dominate the crystal packing, creating a two-dimensional network structure along [011].

## Related literature

For background to lomefloxacin, see: Rubinstein *et al.* (2001 ▶). For related structures, see: Jasinski *et al.* (2009 ▶, 2010*a* ▶,*b* ▶). For puckering parameters, see: Cremer & Pople (1975 ▶). For bond-length data, see: Allen *et al.* (1987 ▶).
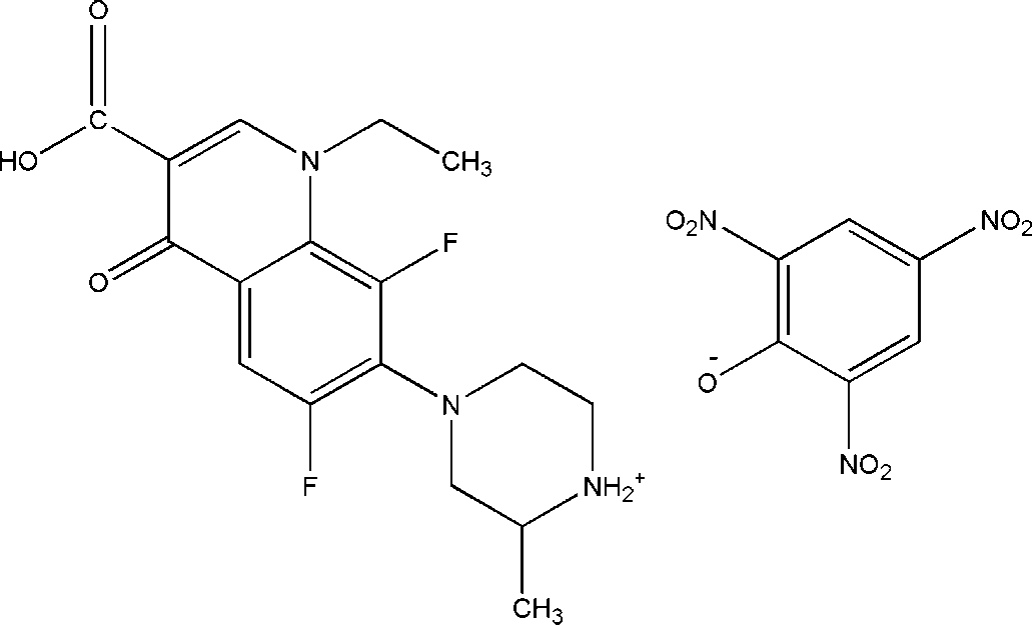


## Experimental

### 

#### Crystal data

C_17_H_20_F_2_N_3_O_3_^+^·C_6_H_2_N_3_O_7_^−^
*M* *_r_* = 580.47
Triclinic, 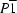

*a* = 10.9314 (4) Å
*b* = 11.6748 (4) Å
*c* = 12.0530 (4) Å
α = 92.969 (3)°
β = 115.555 (3)°
γ = 109.852 (3)°
*V* = 1269.14 (8) Å^3^
*Z* = 2
Cu *K*α radiation
μ = 1.13 mm^−1^
*T* = 123 K
0.44 × 0.33 × 0.19 mm

#### Data collection

Oxford Diffraction Xcalibur Ruby Gemini diffractometer
Absorption correction: multi-scan (*CrysAlis RED*; Oxford Diffraction, 2007 ▶) *T*_min_ = 0.838, *T*_max_ = 1.000
8890 measured reflections
5002 independent reflections
4423 reflections with *I* > 2σ(*I*)
*R*_int_ = 0.018

#### Refinement

*R*[*F* ^2^ > 2σ(*F* ^2^)] = 0.068
*wR*(*F* ^2^) = 0.198
*S* = 1.06
5002 reflections
373 parameters
H-atom parameters constrained
Δρ_max_ = 0.54 e Å^−3^
Δρ_min_ = −0.59 e Å^−3^

### 

Data collection: *CrysAlis PRO* (Oxford Diffraction, 2007 ▶); cell refinement: *CrysAlis PRO*; data reduction: *CrysAlis RED* (Oxford Diffraction, 2007 ▶); program(s) used to solve structure: *SHELXS97* (Sheldrick, 2008 ▶); program(s) used to refine structure: *SHELXL97* (Sheldrick, 2008 ▶); molecular graphics: *SHELXTL* (Sheldrick, 2008 ▶); software used to prepare material for publication: *SHELXTL*.

## Supplementary Material

Crystal structure: contains datablocks global, I. DOI: 10.1107/S1600536811002534/fl2332sup1.cif

Structure factors: contains datablocks I. DOI: 10.1107/S1600536811002534/fl2332Isup2.hkl

Additional supplementary materials:  crystallographic information; 3D view; checkCIF report

## Figures and Tables

**Table 1:** Hydrogen-bond geometry (Å, °)

*D*—H⋯*A*	*D*—H	H⋯*A*	*D*⋯*A*	*D*—H⋯*A*
O2—H2⋯O3	0.84	1.74	2.520 (3)	153
N3—H3*A*⋯O2^i^	0.92	2.09	2.984 (3)	164
N3—H3*B*⋯O1*B*	0.92	1.81	2.714 (3)	166
N3—H3*B*⋯O2*B*	0.92	2.52	2.993 (3)	113

Symmetry code: (i) 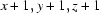.

**Fig. 1.**
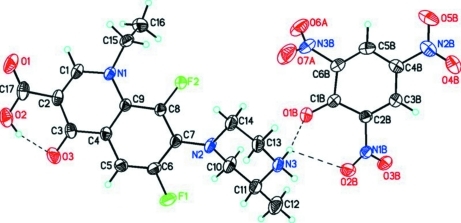
Molecular structure of the title compound showing the atom labeling scheme and 50% probability displacement ellipsoids. Dashed lines indicate an intramolecular O—H···O hydrogen bond in the cation. and a bifurcated N—H···(O,O) intermolecular three-centered hydrogen bond formed between the protonated N atom from the lomefloxacinium cation and the picrate anion. In the picrate anion only the predominate disordered O6A and O7A (0.762 (4)) atoms are displayed.

**Fig. 2.**
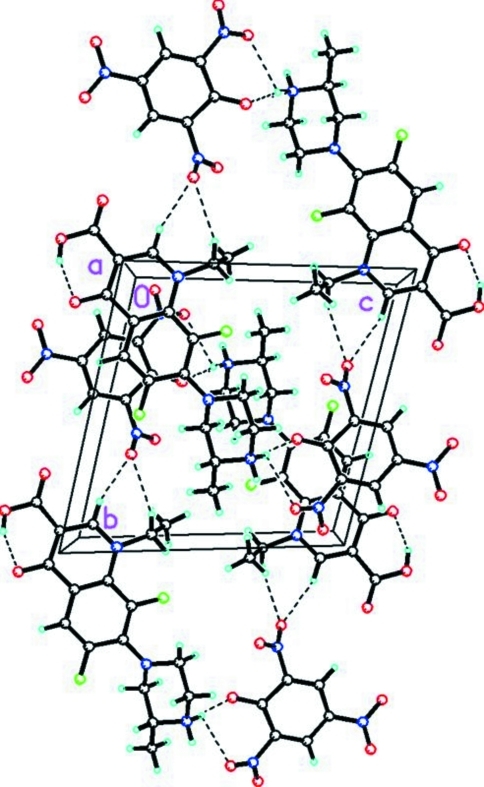
Packing diagram of the title compound viewed down the a axis. Dashed lines indicate N—H···O hydrogen bonds creating a 2-D network along [011].

## supplementary crystallographic information

### Comment

Lomefloxacin hydrochloride is a fluoroquinolone antibiotic used to treat bacterial infections including bronchitis and urinary tract infections. It is also used to prevent urinary tract infections prior to surgery. Lomefloxacin, chemically (*RS*)-1-ethyl-6,8-difluoro-7-(3-methylpiperazin- 1-yl)-4-oxo-quinoline-3-carboxylic acid, is associated with phototoxicity and adverse central nervous system adverse effects (Rubinstein *et al.*, 2001). Recently, the crystal structures of propiverine picrate (Jasinski *et al.*, 2009), imatinibium dipicrate (Jasinski *et al.*, 2010*a*) and chlorimipraminium picrate (Jasinski *et al.*, 2010*b*) have been reported. In continuation of our work on picrates of biologically active compounds, this paper reports the crystal structure of (I), obtained by the interaction of picric acid and lomefloxacin.

In the crystal structure of the title compound, there is one cation-anion pair in the asymmetric unit (Fig. 1). One N atom in the 6-membered piperazine ring (N2/C10/C11/N3/C13/C14) in the lomefloxacinium cation is protonated which adopts a slightly distorted chair conformation with puckering parameters Q, θ and φ of 0.565 (3) Å, 178.0 (3)° and 109 (58)° (Cremer & Pople, 1975). The dihedral angle between the mean planes of the piperazine ring (N2/C10/C11/N3/C13/C14) and the 10-atom ring system of the quinolone group is 43.3 (5)°. The picrate anion interacts with the protonated N atom of an adjacent cation through a bifurcated N—H···O three-center hydrogen bond.  The dihedral angle between the mean planes of the anion benzene and cation piperizine and quinoline rings is 46.2 (9)° and 7.2 (2)°, respectively. The mean planes of the two *o*-NO_2_ and single *p*-NO_2_ groups in the picrate anion are twisted by, 37.9 (5)°, 57.0 (8)° [using predominant component (0.743 (4), O6A & O7A, of disordered O atoms] and 2.5 (1)° with respect to the mean planes of the 6-membered benzene ring. Bond distances (Allen *et al.*, 1987) and angles are in normal ranges. Strong N—H···O hydrogen bonds in concert with weak π–π stacking interactions (Table 2) dominate the crystal packing creating a 2-D network along [011] (Fig. 2).

### Experimental

Lomefloxacin hydrochloride (3.87 g, 0.01 mol) of picric acid (2.99 g, 0.01 mol) was dissolved in 15 ml of dimethyl formamide. The solution was stirred for 15 min over a heating magnetic stirrer at 335 K.  The resulting solution was kept aside at room temperature.  X-ray quality crystals were grown from slow evaporation of dimethyl formamide solution (m.p.: 489 – 491 K).

### Refinement

The O atoms on one of the *o*-nitrate groups in the picrate anion are disordered [occupancy O6A and O7A = 0.762 (4); O6B and O7B = 0.238 (4)]. The N3B–O6A and N3B–O6B dstances were fixed at 1.23Å. The O6A–O7A and O6B–O7B angular distances were fixed at 2.15Å. All of the H atoms were placed in their calculated positions and then refined using the riding model with Atom—H lengths of 0.95Å (CH), 0.99Å (CH_2_), 0.98Å (CH_3_), 0.92Å (NH), or 0.84 (OH). Isotropic displacement parameters for these atoms were set to 1.20 times (NH), 1.45 (OH), 1.19-1.20 (CH, CH_2_) or 1.49 (CH_3_) times *U*_eq_ of the parent atom.

### Crystal data

**Table:**

C_17_H_20_F_2_N_3_O_3_^+^·C_6_H_2_N_3_O_7_^−^	*Z* = 2
*M**_r_* = 580.47	*F*(000) = 600
Triclinic, *P*1	*D*_x_ = 1.519 Mg m^−^^3^
Hall symbol: -P 1	Cu *K*α radiation, λ = 1.54178 Å
*a* = 10.9314 (4) Å	Cell parameters from 6216 reflections
*b* = 11.6748 (4) Å	θ = 4.7–73.8°
*c* = 12.0530 (4) Å	µ = 1.13 mm^−^^1^
α = 92.969 (3)°	*T* = 123 K
β = 115.555 (3)°	Prism, pale yellow
γ = 109.852 (3)°	0.44 × 0.33 × 0.19 mm
*V* = 1269.14 (8) Å^3^	

### Data collection

**Table:**

Oxford Diffraction Xcalibur Ruby Gemini diffractometer	5002 independent reflections
Radiation source: Enhance (Cu) X-ray Source	4423 reflections with *I* > 2σ(*I*)
graphite	*R*_int_ = 0.018
Detector resolution: 10.5081 pixels mm^-1^	θ_max_ = 74.0°, θ_min_ = 4.7°
ω scans	*h* = −13→13
Absorption correction: multi-scan (*CrysAlis RED*; Oxford Diffraction, 2007)	*k* = −14→14
*T*_min_ = 0.838, *T*_max_ = 1.000	*l* = −15→11
8890 measured reflections	

### Refinement

**Table:**

Refinement on *F*^2^	Primary atom site location: structure-invariant direct methods
Least-squares matrix: full	Secondary atom site location: difference Fourier map
*R*[*F*^2^ > 2σ(*F*^2^)] = 0.068	Hydrogen site location: inferred from neighbouring sites
*wR*(*F*^2^) = 0.198	H-atom parameters constrained
*S* = 1.06	*w* = 1/[σ^2^(*F*_o_^2^) + (0.0848*P*)^2^ + 0.7775*P*] where *P* = (*F*_o_^2^ + 2*F*_c_^2^)/3
5002 reflections	(Δ/σ)_max_ = 0.011
373 parameters	Δρ_max_ = 0.54 e Å^−^^3^
0 restraints	Δρ_min_ = −0.59 e Å^−^^3^

### Special details

**Table:**

Geometry. All e.s.d.'s (except the e.s.d. in the dihedral angle between two l.s. planes) are estimated using the full covariance matrix. The cell e.s.d.'s are taken into account individually in the estimation of e.s.d.'s in distances, angles and torsion angles; correlations between e.s.d.'s in cell parameters are only used when they are defined by crystal symmetry. An approximate (isotropic) treatment of cell e.s.d.'s is used for estimating e.s.d.'s involving l.s. planes.
Refinement. Refinement of *F*^2^ against ALL reflections. The weighted *R*-factor *wR* and goodness of fit *S* are based on *F*^2^, conventional *R*-factors *R* are based on *F*, with *F* set to zero for negative *F*^2^. The threshold expression of *F*^2^ > σ(*F*^2^) is used only for calculating *R*-factors(gt) *etc*. and is not relevant to the choice of reflections for refinement. *R*-factors based on *F*^2^ are statistically about twice as large as those based on *F*, and *R*- factors based on ALL data will be even larger.

### Fractional atomic coordinates and isotropic or equivalent isotropic displacement parameters (Å^2^)

**Table:**

	*x*	*y*	*z*	*U*_iso_*/*U*_eq_	Occ. (<1)
F1	0.6041 (2)	0.51552 (14)	0.14890 (17)	0.0702 (5)	
F2	0.5633 (2)	0.21345 (13)	0.38653 (13)	0.0626 (5)	
O1	0.1693 (3)	−0.2597 (2)	−0.1643 (2)	0.0873 (8)	
O2	0.1665 (3)	−0.1195 (2)	−0.2795 (2)	0.0762 (7)	
H2	0.1948	−0.0413	−0.2670	0.114*	
O3	0.3100 (2)	0.1106 (2)	−0.17445 (17)	0.0677 (6)	
N1	0.4330 (2)	0.01780 (17)	0.16877 (19)	0.0436 (5)	
N2	0.6695 (3)	0.46292 (19)	0.3820 (2)	0.0533 (6)	
N3	0.8631 (2)	0.67661 (19)	0.58301 (19)	0.0496 (5)	
H3A	0.9470	0.7498	0.6208	0.060*	
H3B	0.8207	0.6649	0.6352	0.060*	
C1	0.3493 (3)	−0.0691 (2)	0.0590 (2)	0.0482 (6)	
H1A	0.3202	−0.1545	0.0631	0.058*	
C2	0.3035 (3)	−0.0441 (2)	−0.0569 (2)	0.0495 (6)	
C3	0.3465 (3)	0.0821 (3)	−0.0690 (2)	0.0485 (6)	
C4	0.4355 (2)	0.1778 (2)	0.0492 (2)	0.0418 (5)	
C5	0.4789 (3)	0.3043 (2)	0.0451 (2)	0.0478 (6)	
H5A	0.4542	0.3271	−0.0338	0.057*	
C6	0.5558 (3)	0.3938 (2)	0.1536 (2)	0.0484 (6)	
C7	0.5904 (3)	0.3680 (2)	0.2737 (2)	0.0435 (5)	
C8	0.5437 (3)	0.2414 (2)	0.2746 (2)	0.0430 (5)	
C9	0.4727 (2)	0.1443 (2)	0.1660 (2)	0.0391 (5)	
C10	0.6250 (3)	0.5649 (3)	0.3947 (3)	0.0626 (8)	
H10A	0.5717	0.5482	0.4448	0.075*	
H10B	0.5562	0.5690	0.3098	0.075*	
C11	0.7563 (3)	0.6884 (2)	0.4582 (3)	0.0592 (7)	
H11A	0.8060	0.7051	0.4044	0.071*	
C12	0.7179 (5)	0.7965 (3)	0.4768 (4)	0.0860 (12)	
H12A	0.8083	0.8737	0.5171	0.129*	
H12B	0.6708	0.7830	0.5309	0.129*	
H12C	0.6495	0.8038	0.3946	0.129*	
C13	0.9051 (3)	0.5702 (3)	0.5682 (3)	0.0572 (7)	
H13A	0.9588	0.5861	0.5184	0.069*	
H13B	0.9723	0.5637	0.6526	0.069*	
C14	0.7718 (3)	0.4506 (2)	0.5035 (3)	0.0604 (8)	
H14A	0.8010	0.3818	0.4899	0.072*	
H14B	0.7229	0.4303	0.5567	0.072*	
C15	0.4738 (3)	−0.0307 (2)	0.2860 (3)	0.0547 (6)	
H15A	0.5682	0.0326	0.3549	0.066*	
H15B	0.4897	−0.1073	0.2707	0.066*	
C16	0.3574 (4)	−0.0604 (3)	0.3270 (3)	0.0721 (9)	
H16A	0.3826	−0.1023	0.3970	0.108*	
H16B	0.2612	−0.1157	0.2559	0.108*	
H16C	0.3522	0.0173	0.3550	0.108*	
C17	0.2083 (3)	−0.1516 (3)	−0.1695 (3)	0.0635 (8)	
O1B	0.7431 (2)	0.60714 (18)	0.73716 (18)	0.0620 (5)	
O2B	0.9064 (3)	0.8637 (2)	0.7905 (2)	0.0755 (7)	
O3B	0.8490 (2)	0.94720 (17)	0.9138 (2)	0.0563 (5)	
O4B	1.0085 (4)	0.8183 (3)	1.3141 (2)	0.0968 (9)	
O5B	0.9630 (4)	0.6231 (3)	1.3095 (2)	0.0926 (8)	
O6A	0.6327 (5)	0.3366 (3)	0.8566 (5)	0.1035 (10)	0.762 (4)
O7A	0.7909 (5)	0.3882 (3)	0.7890 (4)	0.1035 (10)	0.762 (4)
O6B	0.6273 (9)	0.3566 (9)	0.7639 (9)	0.1035 (10)	0.238 (4)
O7B	0.8257 (10)	0.3560 (9)	0.9080 (10)	0.1035 (10)	0.238 (4)
N1B	0.8724 (2)	0.86209 (18)	0.8747 (2)	0.0466 (5)	
N2B	0.9639 (3)	0.7109 (3)	1.2577 (3)	0.0683 (7)	
N3B	0.7447 (3)	0.4116 (2)	0.8604 (3)	0.0675 (7)	
C1B	0.7998 (3)	0.6332 (2)	0.8548 (2)	0.0476 (6)	
C2B	0.8633 (3)	0.7566 (2)	0.9345 (2)	0.0443 (5)	
C3B	0.9121 (3)	0.7818 (2)	1.0621 (2)	0.0482 (6)	
H3BA	0.9482	0.8649	1.1084	0.058*	
C4B	0.9077 (3)	0.6843 (3)	1.1220 (3)	0.0544 (6)	
C5B	0.8520 (3)	0.5620 (3)	1.0552 (3)	0.0593 (7)	
H5BA	0.8492	0.4953	1.0972	0.071*	
C6B	0.8019 (3)	0.5399 (2)	0.9289 (3)	0.0544 (6)	

### Atomic displacement parameters (Å^2^)

**Table:**

	*U*^11^	*U*^22^	*U*^33^	*U*^12^	*U*^13^	*U*^23^
F1	0.0779 (11)	0.0377 (8)	0.0661 (10)	0.0129 (8)	0.0173 (9)	0.0181 (7)
F2	0.0898 (12)	0.0390 (8)	0.0360 (7)	0.0139 (8)	0.0201 (7)	0.0050 (6)
O1	0.0778 (16)	0.0513 (13)	0.0785 (16)	0.0096 (11)	0.0083 (12)	−0.0238 (11)
O2	0.0637 (13)	0.0804 (15)	0.0460 (11)	0.0075 (12)	0.0134 (10)	−0.0153 (10)
O3	0.0677 (13)	0.0764 (14)	0.0347 (9)	0.0175 (11)	0.0137 (9)	0.0042 (9)
N1	0.0435 (10)	0.0321 (9)	0.0418 (10)	0.0140 (8)	0.0110 (8)	0.0012 (8)
N2	0.0547 (12)	0.0340 (10)	0.0436 (11)	0.0187 (9)	0.0010 (9)	−0.0036 (8)
N3	0.0522 (12)	0.0372 (10)	0.0396 (10)	0.0137 (9)	0.0098 (9)	−0.0036 (8)
C1	0.0421 (12)	0.0350 (11)	0.0525 (14)	0.0124 (10)	0.0141 (11)	−0.0045 (10)
C2	0.0391 (12)	0.0476 (14)	0.0442 (13)	0.0125 (10)	0.0111 (10)	−0.0091 (10)
C3	0.0392 (12)	0.0571 (15)	0.0373 (12)	0.0158 (11)	0.0123 (10)	−0.0010 (10)
C4	0.0376 (11)	0.0427 (12)	0.0350 (11)	0.0142 (9)	0.0111 (9)	0.0023 (9)
C5	0.0461 (13)	0.0479 (13)	0.0395 (12)	0.0169 (11)	0.0133 (10)	0.0124 (10)
C6	0.0460 (13)	0.0344 (11)	0.0507 (14)	0.0123 (10)	0.0139 (11)	0.0123 (10)
C7	0.0384 (11)	0.0345 (11)	0.0399 (12)	0.0117 (9)	0.0069 (9)	0.0007 (9)
C8	0.0448 (12)	0.0368 (11)	0.0331 (11)	0.0127 (9)	0.0098 (9)	0.0037 (9)
C9	0.0371 (11)	0.0337 (11)	0.0377 (11)	0.0129 (9)	0.0120 (9)	0.0033 (9)
C10	0.0559 (15)	0.0452 (14)	0.0604 (16)	0.0235 (12)	0.0049 (13)	−0.0038 (12)
C11	0.0684 (17)	0.0419 (13)	0.0485 (14)	0.0221 (13)	0.0131 (13)	0.0025 (11)
C12	0.109 (3)	0.0548 (18)	0.071 (2)	0.046 (2)	0.015 (2)	0.0017 (15)
C13	0.0565 (15)	0.0496 (14)	0.0442 (13)	0.0240 (12)	0.0056 (11)	−0.0034 (11)
C14	0.0648 (17)	0.0407 (13)	0.0462 (14)	0.0229 (12)	0.0017 (12)	−0.0015 (11)
C15	0.0656 (16)	0.0363 (12)	0.0500 (14)	0.0222 (12)	0.0160 (12)	0.0106 (10)
C16	0.082 (2)	0.0591 (17)	0.0626 (18)	0.0180 (16)	0.0319 (17)	0.0185 (14)
C17	0.0458 (14)	0.0654 (19)	0.0528 (16)	0.0132 (13)	0.0113 (12)	−0.0158 (13)
O1B	0.0670 (12)	0.0486 (10)	0.0480 (10)	0.0090 (9)	0.0207 (9)	0.0009 (8)
O2B	0.128 (2)	0.0528 (12)	0.0654 (13)	0.0362 (13)	0.0615 (14)	0.0211 (10)
O3B	0.0553 (10)	0.0428 (9)	0.0721 (12)	0.0245 (8)	0.0280 (9)	0.0123 (8)
O4B	0.126 (2)	0.100 (2)	0.0534 (13)	0.0370 (18)	0.0404 (15)	0.0133 (13)
O5B	0.122 (2)	0.119 (2)	0.0731 (15)	0.0732 (19)	0.0545 (16)	0.0567 (16)
O6A	0.123 (2)	0.0541 (13)	0.137 (3)	0.0198 (13)	0.080 (2)	0.0015 (14)
O7A	0.123 (2)	0.0541 (13)	0.137 (3)	0.0198 (13)	0.080 (2)	0.0015 (14)
O6B	0.123 (2)	0.0541 (13)	0.137 (3)	0.0198 (13)	0.080 (2)	0.0015 (14)
O7B	0.123 (2)	0.0541 (13)	0.137 (3)	0.0198 (13)	0.080 (2)	0.0015 (14)
N1B	0.0487 (11)	0.0368 (10)	0.0450 (11)	0.0152 (9)	0.0163 (9)	0.0070 (8)
N2B	0.0743 (17)	0.087 (2)	0.0550 (14)	0.0398 (15)	0.0343 (13)	0.0262 (14)
N3B	0.0766 (17)	0.0369 (12)	0.0908 (19)	0.0209 (12)	0.0430 (15)	0.0145 (12)
C1B	0.0467 (13)	0.0384 (12)	0.0545 (14)	0.0144 (10)	0.0239 (11)	0.0086 (10)
C2B	0.0450 (12)	0.0389 (12)	0.0475 (13)	0.0167 (10)	0.0208 (10)	0.0114 (10)
C3B	0.0479 (13)	0.0458 (13)	0.0495 (13)	0.0180 (11)	0.0229 (11)	0.0088 (10)
C4B	0.0588 (15)	0.0616 (16)	0.0507 (14)	0.0279 (13)	0.0291 (13)	0.0209 (12)
C5B	0.0689 (17)	0.0520 (15)	0.0729 (19)	0.0294 (14)	0.0420 (15)	0.0291 (14)
C6B	0.0562 (15)	0.0409 (13)	0.0667 (17)	0.0184 (11)	0.0306 (13)	0.0138 (12)

### Geometric parameters (Å, °)

**Table:**

F1—C6	1.351 (3)	C12—H12B	0.9800
F2—C8	1.348 (3)	C12—H12C	0.9800
O1—C17	1.202 (4)	C13—C14	1.485 (4)
O2—C17	1.323 (4)	C13—H13A	0.9900
O2—H2	0.8400	C13—H13B	0.9900
O3—C3	1.258 (3)	C14—H14A	0.9900
N1—C1	1.343 (3)	C14—H14B	0.9900
N1—C9	1.397 (3)	C15—C16	1.499 (5)
N1—C15	1.492 (3)	C15—H15A	0.9900
N2—C7	1.381 (3)	C15—H15B	0.9900
N2—C10	1.455 (3)	C16—H16A	0.9800
N2—C14	1.460 (3)	C16—H16B	0.9800
N3—C13	1.490 (3)	C16—H16C	0.9800
N3—C11	1.504 (3)	O1B—C1B	1.248 (3)
N3—H3A	0.9200	O2B—N1B	1.221 (3)
N3—H3B	0.9200	O3B—N1B	1.226 (3)
C1—C2	1.351 (4)	O4B—N2B	1.218 (4)
C1—H1A	0.9500	O5B—N2B	1.227 (4)
C2—C3	1.422 (4)	O6A—N3B	1.219 (3)
C2—C17	1.489 (3)	O7A—N3B	1.2298 (19)
C3—C4	1.459 (3)	O6B—N3B	1.219 (5)
C4—C5	1.400 (4)	O7B—N3B	1.234 (2)
C4—C9	1.405 (3)	N1B—C2B	1.455 (3)
C5—C6	1.350 (4)	N2B—C4B	1.449 (4)
C5—H5A	0.9500	N3B—C6B	1.451 (3)
C6—C7	1.407 (4)	C1B—C2B	1.443 (3)
C7—C8	1.392 (3)	C1B—C6B	1.444 (4)
C8—C9	1.405 (3)	C2B—C3B	1.370 (4)
C10—C11	1.503 (4)	C3B—C4B	1.377 (4)
C10—H10A	0.9900	C3B—H3BA	0.9500
C10—H10B	0.9900	C4B—C5B	1.388 (4)
C11—C12	1.493 (4)	C5B—C6B	1.351 (4)
C11—H11A	1.0000	C5B—H5BA	0.9500
C12—H12A	0.9800		
			
C17—O2—H2	109.5	C14—C13—N3	110.8 (2)
C1—N1—C9	119.0 (2)	C14—C13—H13A	109.5
C1—N1—C15	116.0 (2)	N3—C13—H13A	109.5
C9—N1—C15	125.02 (19)	C14—C13—H13B	109.5
C7—N2—C10	121.8 (2)	N3—C13—H13B	109.5
C7—N2—C14	122.7 (2)	H13A—C13—H13B	108.1
C10—N2—C14	112.8 (2)	N2—C14—C13	109.3 (2)
C13—N3—C11	112.07 (19)	N2—C14—H14A	109.8
C13—N3—H3A	109.2	C13—C14—H14A	109.8
C11—N3—H3A	109.2	N2—C14—H14B	109.8
C13—N3—H3B	109.2	C13—C14—H14B	109.8
C11—N3—H3B	109.2	H14A—C14—H14B	108.3
H3A—N3—H3B	107.9	N1—C15—C16	112.5 (2)
N1—C1—C2	124.9 (2)	N1—C15—H15A	109.1
N1—C1—H1A	117.5	C16—C15—H15A	109.1
C2—C1—H1A	117.5	N1—C15—H15B	109.1
C1—C2—C3	120.0 (2)	C16—C15—H15B	109.1
C1—C2—C17	118.2 (3)	H15A—C15—H15B	107.8
C3—C2—C17	121.8 (3)	C15—C16—H16A	109.5
O3—C3—C2	122.7 (2)	C15—C16—H16B	109.5
O3—C3—C4	121.5 (3)	H16A—C16—H16B	109.5
C2—C3—C4	115.8 (2)	C15—C16—H16C	109.5
C5—C4—C9	120.0 (2)	H16A—C16—H16C	109.5
C5—C4—C3	119.2 (2)	H16B—C16—H16C	109.5
C9—C4—C3	120.7 (2)	O1—C17—O2	121.1 (3)
C6—C5—C4	119.9 (2)	O1—C17—C2	124.3 (3)
C6—C5—H5A	120.1	O2—C17—C2	114.5 (3)
C4—C5—H5A	120.1	O2B—N1B—O3B	122.9 (2)
C5—C6—F1	119.3 (2)	O2B—N1B—C2B	118.9 (2)
C5—C6—C7	123.6 (2)	O3B—N1B—C2B	118.1 (2)
F1—C6—C7	117.2 (2)	O4B—N2B—O5B	123.5 (3)
N2—C7—C8	123.3 (2)	O4B—N2B—C4B	118.7 (3)
N2—C7—C6	121.4 (2)	O5B—N2B—C4B	117.9 (3)
C8—C7—C6	115.2 (2)	O6A—N3B—O7A	121.6 (3)
F2—C8—C7	116.6 (2)	O6B—N3B—O7B	119.2 (6)
F2—C8—C9	119.6 (2)	O6B—N3B—C6B	126.3 (5)
C7—C8—C9	123.8 (2)	O6A—N3B—C6B	117.5 (3)
N1—C9—C4	119.3 (2)	O7A—N3B—C6B	119.2 (3)
N1—C9—C8	123.4 (2)	O7B—N3B—C6B	114.5 (5)
C4—C9—C8	117.2 (2)	O1B—C1B—C2B	125.8 (2)
N2—C10—C11	111.5 (2)	O1B—C1B—C6B	123.3 (2)
N2—C10—H10A	109.3	C2B—C1B—C6B	110.8 (2)
C11—C10—H10A	109.3	C3B—C2B—C1B	124.9 (2)
N2—C10—H10B	109.3	C3B—C2B—N1B	117.3 (2)
C11—C10—H10B	109.3	C1B—C2B—N1B	117.8 (2)
H10A—C10—H10B	108.0	C2B—C3B—C4B	118.7 (2)
C12—C11—C10	114.0 (3)	C2B—C3B—H3BA	120.7
C12—C11—N3	110.5 (2)	C4B—C3B—H3BA	120.7
C10—C11—N3	108.1 (2)	C3B—C4B—C5B	121.3 (3)
C12—C11—H11A	108.0	C3B—C4B—N2B	119.0 (3)
C10—C11—H11A	108.0	C5B—C4B—N2B	119.7 (3)
N3—C11—H11A	108.0	C6B—C5B—C4B	118.5 (3)
C11—C12—H12A	109.5	C6B—C5B—H5BA	120.7
C11—C12—H12B	109.5	C4B—C5B—H5BA	120.7
H12A—C12—H12B	109.5	C5B—C6B—C1B	125.7 (3)
C11—C12—H12C	109.5	C5B—C6B—N3B	117.9 (3)
H12A—C12—H12C	109.5	C1B—C6B—N3B	116.4 (3)
H12B—C12—H12C	109.5		
			
C9—N1—C1—C2	2.6 (4)	C13—N3—C11—C12	−179.5 (3)
C15—N1—C1—C2	−178.6 (2)	C13—N3—C11—C10	55.1 (3)
N1—C1—C2—C3	1.4 (4)	C11—N3—C13—C14	−57.0 (3)
N1—C1—C2—C17	−178.7 (2)	C7—N2—C14—C13	140.2 (3)
C1—C2—C3—O3	178.3 (2)	C10—N2—C14—C13	−57.9 (4)
C17—C2—C3—O3	−1.6 (4)	N3—C13—C14—N2	56.2 (3)
C1—C2—C3—C4	−2.2 (4)	C1—N1—C15—C16	−85.8 (3)
C17—C2—C3—C4	177.9 (2)	C9—N1—C15—C16	92.9 (3)
O3—C3—C4—C5	1.2 (4)	C1—C2—C17—O1	−0.1 (4)
C2—C3—C4—C5	−178.3 (2)	C3—C2—C17—O1	179.8 (3)
O3—C3—C4—C9	178.6 (2)	C1—C2—C17—O2	179.2 (3)
C2—C3—C4—C9	−0.9 (3)	C3—C2—C17—O2	−0.9 (4)
C9—C4—C5—C6	−0.3 (4)	O1B—C1B—C2B—C3B	173.3 (3)
C3—C4—C5—C6	177.1 (2)	C6B—C1B—C2B—C3B	−3.2 (4)
C4—C5—C6—F1	176.3 (2)	O1B—C1B—C2B—N1B	−4.6 (4)
C4—C5—C6—C7	−3.2 (4)	C6B—C1B—C2B—N1B	178.8 (2)
C10—N2—C7—C8	−129.4 (3)	O2B—N1B—C2B—C3B	142.1 (3)
C14—N2—C7—C8	30.8 (4)	O3B—N1B—C2B—C3B	−36.0 (3)
C10—N2—C7—C6	52.8 (4)	O2B—N1B—C2B—C1B	−39.8 (3)
C14—N2—C7—C6	−146.9 (3)	O3B—N1B—C2B—C1B	142.1 (2)
C5—C6—C7—N2	180.0 (2)	C1B—C2B—C3B—C4B	3.3 (4)
F1—C6—C7—N2	0.4 (4)	N1B—C2B—C3B—C4B	−178.8 (2)
C5—C6—C7—C8	2.0 (4)	C2B—C3B—C4B—C5B	−1.6 (4)
F1—C6—C7—C8	−177.5 (2)	C2B—C3B—C4B—N2B	177.8 (2)
N2—C7—C8—F2	8.0 (4)	O4B—N2B—C4B—C3B	2.6 (4)
C6—C7—C8—F2	−174.1 (2)	O5B—N2B—C4B—C3B	−177.9 (3)
N2—C7—C8—C9	−175.2 (2)	O4B—N2B—C4B—C5B	−178.0 (3)
C6—C7—C8—C9	2.7 (4)	O5B—N2B—C4B—C5B	1.6 (4)
C1—N1—C9—C4	−5.6 (3)	C3B—C4B—C5B—C6B	0.2 (4)
C15—N1—C9—C4	175.7 (2)	N2B—C4B—C5B—C6B	−179.2 (3)
C1—N1—C9—C8	171.8 (2)	C4B—C5B—C6B—C1B	−0.4 (5)
C15—N1—C9—C8	−6.9 (4)	C4B—C5B—C6B—N3B	179.7 (3)
C5—C4—C9—N1	−177.8 (2)	O1B—C1B—C6B—C5B	−174.9 (3)
C3—C4—C9—N1	4.8 (3)	C2B—C1B—C6B—C5B	1.8 (4)
C5—C4—C9—C8	4.6 (3)	O1B—C1B—C6B—N3B	5.0 (4)
C3—C4—C9—C8	−172.8 (2)	C2B—C1B—C6B—N3B	−178.3 (2)
F2—C8—C9—N1	−6.6 (4)	O6B—N3B—C6B—C5B	130.8 (9)
C7—C8—C9—N1	176.6 (2)	O6A—N3B—C6B—C5B	65.1 (5)
F2—C8—C9—C4	170.8 (2)	O7A—N3B—C6B—C5B	−129.3 (4)
C7—C8—C9—C4	−5.9 (4)	O7B—N3B—C6B—C5B	−48.8 (7)
C7—N2—C10—C11	−139.1 (3)	O6B—N3B—C6B—C1B	−49.1 (9)
C14—N2—C10—C11	58.8 (4)	O6A—N3B—C6B—C1B	−114.8 (4)
N2—C10—C11—C12	−178.6 (3)	O7A—N3B—C6B—C1B	50.8 (5)
N2—C10—C11—N3	−55.3 (3)	O7B—N3B—C6B—C1B	131.3 (7)

### Hydrogen-bond geometry (Å, °)

**Table:**

*D*—H···*A*	*D*—H	H···*A*	*D*···*A*	*D*—H···*A*
O2—H2···O3	0.84	1.74	2.520 (3)	153
N3—H3A···O2^i^	0.92	2.09	2.984 (3)	164
N3—H3B···O1B	0.92	1.81	2.714 (3)	166
N3—H3B···O2B	0.92	2.52	2.993 (3)	113

Symmetry codes: (i) *x*+1, *y*+1, *z*+1.

### Table 2 Cg···Cg π stacking interactions, Cg1 is the centroid of ring N1/C1/C2/C3/C4/C9; [Symmetry code: (i) 1-x, -y, -z]

**Table:**

*Cg*I···*Cg*J	Cg···Cg (Å)	CgI Perp (Å)	Cgj Perp (Å)	Slippage (Å)
Cg1···Cg1^i^	3.6460 (14)	3.3385 (10)	3.3385 (10)	1.46 (6)
